# Oral tolerance as antigen-specific immunotherapy

**DOI:** 10.1093/immadv/ltab017

**Published:** 2021-08-25

**Authors:** Natália Pinheiro-Rosa, Lícia Torres, Mariana de Almeida Oliveira, Marcos Felipe Andrade-Oliveira, Mauro Andrade de Freitas Guimarães, Monique Macedo Coelho, Juliana de Lima Alves, Tatiani Uceli Maioli, Ana M Caetano Faria

**Affiliations:** 1 Departamento de Bioquímica e Imunologia, Instituto de Ciências Biológicas, Universidade Federal de Minas Gerais, Belo Horizonte, MG, Brazil; 2 Departamento de Nutrição, Escola de Enfermagem, Universidade Federal de Minas Gerais, Belo Horizonte, MG, Brazil

**Keywords:** oral tolerance, gut mucosa, oral immunotherapy, regulatory T cells

## Abstract

Oral tolerance is a physiological phenomenon described more than a century ago as a suppressive immune response to antigens that gain access to the body by the oral route. It is a robust and long-lasting event with local and systemic effects in which the generation of mucosally induced regulatory T cells (iTreg) plays an essential role. The idea of using oral tolerance to inhibit autoimmune and allergic diseases by oral administration of target antigens was an important development that was successfully tested in 1980s. Since then, several studies have shown that feeding specific antigens can be used to prevent and control chronic inflammatory diseases in both animal models and clinically. Therefore, oral tolerance can be classified as an antigen-specific form of oral immunotherapy (OIT). In the light of novel findings on mechanisms, sites of induction and factors affecting oral tolerance, this review will focus on specific characteristics of oral tolerance induction and how they impact in its therapeutic application.

## Introduction

Oral tolerance is described as a physiological phenomenon that contributes to prevent pathological conditions to food protein and commensal microbiota, inhibiting responses that could cause damage such as hypersensitivity reactions, lymphocyte proliferation, and antibody formation [1]. Most of all, tolerance induced to these natural antigens that reach the intestinal lumen seems to be an analog of tolerance to self-components. Since microbiota and dietary antigens are part of our physiological interface with the external environment, tolerogenic mechanisms in the mucosal surfaces must have evolved to treat these antigens as complementary or quasi-self.

The first reports on oral tolerance go back to the beginning of 20th century when Alexander Besredka showed that guinea pigs that ingested milk became refractory to anaphylaxis induced by intracerebral injection of milk [2]. In 1911, Wells and Osborne also observed that guinea pigs that where fed a corn-containing diet fail to develop anaphylaxis to the corn protein zein [3]. Subsequent studies in 1946 and later in 1970s characterized the phenomenon as an immunological event involving the action of suppressor T cells [4]. However, it was only in the late 1980s that pioneer studies using animal models of autoimmune diseases showed the potential clinical use of the oral route to inhibit inflammatory diseases. Reports from four different groups showed that feeding autoantigens such as collagen and myelin basic protein (MBP) could prevent the development of experimental models of arthritis [5, 6] and multiple sclerosis [7, 8] respectively. Oral tolerance in humans was demonstrated in 1994 by Husby and colleagues who showed that ingestion of keyhole limpet hemocyanin by adults prevented the development of delayed hypersensitivity reaction to the fed antigen [9]. After these observations, several pre-clinical and clinical studies investigated in more detail the potential use of oral administration of target antigens as a therapeutic alternative to attenuate clinical signs of allergies, multiple sclerosis, rheumatoid arthritis, uveitis, and other inflammatory diseases [4, 10].

More recently, a concept named oral immunotherapy (OIT) has been proposed to describe the use of the oral route to modulate inflammatory conditions such as allergic and autoimmune diseases by inducing specific and non-specific immunoregulatory mechanisms. OIT includes the use of a broad range of modulatory agents. Some of them are non-specific effector molecules such as interleukin 10 (IL-10), IL-4, or anti-CD3 monoclonal antibodies that act improving the proliferation and function of regulatory T cells in the gut [11–13]. Many other OIT studies focus on the oral delivery of target antigens to trigger specific suppression of allergic and autoimmune diseases [10]. The latter describes better what was originally defined as oral tolerance induction since immunological tolerance relies on activation of regulatory mechanisms affecting specific undesirable inflammatory immune responses. This is an important distinction since one of the major advantages of oral tolerance is the fact that it does not cause any degree of immunosuppression keeping intact the immune response to other potentially harmful antigens [14]. On the other hand, oral administration of some immunologically active molecules such as IL-10, IL-4, and anti-CD3 in combination with specific antigens enhances the suppressive effect of oral tolerance [11–13].

Other forms of immunotherapy have been reported using the nasal, sublingual and epidermic routes with variable results [15]. A distinctive feature of the oral route is the large surface of contact of the gut mucosa and the multiple immunoregulatory elements that it lodges. As already well reviewed by others [15–17], broad spectrum OIT and antigen-induced oral tolerance approaches explore the privileged tolerogenic milieu of the intestinal mucosa for therapeutic purposes.

Several aspects of oral and mucosal tolerance have been already extensively reviewed by others. We will focus our attention on the mechanisms and issues that are relevant for the therapeutic use of oral tolerance as antigen-specific OIT.

## Oral tolerance as a mechanism for intestinal homeostasis and systemic regulation

### Sites of oral tolerance induction

The organs and sites involved in iTreg cell generation and oral tolerance induction are relevant not only for our better understanding of the physiological immune responses to luminal antigens but also to help design strategies to use these responses for therapeutic purposes.

The intestine is the largest contact surface between the organism and the external environment, and in addition, the gut-associated lymphoid tissue (GALT) is the most complex and diverse within the immune system, with many types of cells and structures. The GALT contains around 10^12^ lymphoid cells, and it is continuously exposed to a large amount of antigens. Between 130 and 190 g of protein reach the gut continuously [18], and about 10^12^/cm^3^ commensal bacteria (microbiota) inhabit the human intestinal lumen [19], with the abundance increasing in the most distal parts of the colon. Immune cells in the gut mucosa face a continuous challenge since they are also stimulated by a plethora of pathogenic and toxic agents, and their adaptation to the intestinal environment requires constant discrimination between natural stimulation coming from dietary antigens and autochthonous microbiota from pathogens that need to be cleared. Inflammatory gut immunity is required to clear intestinal infectious agents whereas oral tolerance is the physiological response to the harmless natural antigens at steady state. Two important questions that arise on studying these dichotomous responses are: (1) how immune cells and structures in the gut mucosa manage to mount such dichotomous responses; (2) whether the tolerogenic responses generated by microbiota and dietary antigens are limited to control local inflammation and preserve gut homeostasis or whether they also yield systemic responses.

Since the gut-draining lymph nodes (gLN) are critical sites that orchestrate the immune responses to luminal antigens, a recent study showed evidence that these lymph nodes are immunologically distinct [20, 21] and support different immune responses depending on the intestinal segment that they drain [20]. Proximal gLNs that drain the duodenum and jejunum where dietary proteins are absorbed and processed, host higher frequencies of tolerogenic CD103^+^CD11b^-^ conventional dendritic cells (cDCs) and induces CD4+ Forkhead box p3 (Foxp3)^+^ induced regulatory T (iTregs) cells upon oral antigen administration [20]. On the other hand, distal gLNs that drain ileum and colon where most of gut microbiota is located, harbor high frequencies of pro-inflammatory cDCs, effector T_H_17 lymphocytes and RAR-related orphan receptor gamma t (RORγt)^+^ iTregs upon ileal antigen challenge. Therefore, proximal gut lymph nodes favor tolerance induction, while distal gut lymph nodes favor inflammatory responses [20]. This lymphatic compartmentalization mirrors the gut functional activities and avoids local immunological conflict; it allows the intestine to handle colonic infection while securing tolerance to ingested antigens. This report is in line with several other studies demonstrating the key role of mesenteric lymph nodes (mLNs), which are part of the gLN, in oral tolerance induction [1, 22, 23].

As already mentioned, one of the most important feature of oral tolerance is its systemic effect. It has been proposed that the response to protein antigens in the small intestine is capable of generating both local and systemic tolerance, while the stimulus generated by the colon microbiota leads mainly to intestinal homeostasis [16]. A critical question on the systemic effects of oral tolerance is how immune responses in other sites of the body are inhibited by feeding antigens. Some studies suggest that dietary antigens reach the blood, and that the liver would participate in the induction of a type of intravenous tolerance. Indeed, intestinal antigens reach this organ via the portal vein before entering in the systemic circulation [16]. Injection of antigens in portal vein has been shown to prevent contact hypersensitivity response [24], delay-type hypersensitivity response [25], and to improve prognoses in surgical brain injury [26] in mice. CD4^+^CD25^+^Foxp3^+^ iTreg cell can be generated during liver-induced tolerance and in vivo depletion of plasmocitoid DCs (pDCs), which are enriched in the liver, by monoclonal antibodies results in impairment of oral tolerance induction [27]. Liver transplantation results in systemic donor-specific T-cell tolerance and portal vein administered antigens generate systemic suppression by many mechanisms as already reviewed [28, 29]. Others have shown that antigen feeding can activate specific T cells for dietary antigens also in peripheral lymph nodes, not just in the mesenteric ones [29], and that serum of mice that recently received ovalbumin (OVA) orally can prevent the induction of delayed-type hypersensitivity when transferred intraperitoneally to naive recipients [30].

Despite reaching the systemic circulation, fed antigens do not rely on systemic lymphoid organs such as spleen for oral tolerance induction since splenectomized mice can be rendered tolerant by the oral route (non-published results from our group). Interestingly, even lymphoid structures in the gut such as Peyer patches (PP) are also not essential for oral tolerance induction. The progeny of mice that were treated with LTβ-receptor-IgG-fusion-protein during pregnancy and do not form PP could also be orally tolerized [31]. The role of lymphoid structures in the human oral cavity such as the Waldeyer’s ring in tolerance induction using sublingual administration of antigens have also been recently investigated. The Waldeyer’s ring includes nasopharyngeal tonsils (adenoids), tubal, palatine and lingual tonsils. Animal studies suggest that the lingual tonsil can be considered as an inductive site sampling and processing antigens to stimulate naive T and B lymphocytes. Sublingual delivery of antigens to mice can inhibit Th2 cells and allergic responses by a variety of mechanisms including the development of regulatory B cells (Bregs) and iTregs that secrete IL-10, transforming growth factor β (TGF-β) and IL-35 in the lingual tonsils [32]. In humans, sublingual immunotherapy (SLIT) has been shown to inhibit allergic rhinitis to allergens such as grass polen and birch polen associated with the induction of CD4+Foxp3+ and IL-10+ iTregs in lingual tonsils [32]. However, differences in the mechanisms triggered by sublingual versus oral delivered antigens exist [33] and oral tolerance does not depend on lymphoid structures in the oral cavity since countless studies report successful inhibition of inflammatory responses by intragastric administration of antigen (gavage) [10, 16].

**Figure 1.**
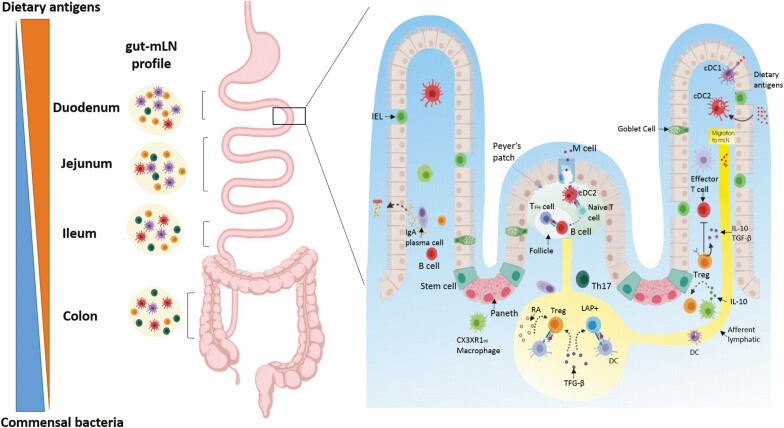
Mechanisms of oral tolerance induction. The contact with dietary antigens decreases from proximal to the distal parts of the intestine and the numbers of bacteria increase in distal segments. In addition, gut lymph nodes (gLNs) draining different gut segments are immunologically distinct and adapted to the region they drain. Duodenal (D)-gLNs have high frequencies of Foxp3+ Tregs and tolerogenic cDCs when compared with distal gLNs. cDCs derived from the LP and proximal gLNs produce large amounts of RA, TGF-β and present a high expression of Aldh1a2. Production of RA by the DCs during interaction with T cells in the presence of TGF-β induces the expression of CCR9 and α4β7, converts naive T cells into Foxp3+ iTregs, while suppressing differentiation of TGFβ-dependent Th17 cells. Conversely, distal gLN (C-gLN, I-gLN, J-gLN) harbor high frequencies of Th17 and RORγT+ iTreg cells at steady state being less tolerogenic. Antigen uptake occurs through a variety of mechanisms, including transport of the antigens across M cells in Peyer’s patches (PP), by DCs that capture antigens associated with goblet cells, indirectly through villi epithelial cells or after antigen transfer from CX3CR1 macrophages that uptake luminal antigens. CCR7+CD103+ DCs are more efficient in inducing iTregs and tolerance upon migration to gLNs carrying dietary antigens. The presence of TGFβ induces latency-associated peptide LAP+ Tregs by action of αvβ8 integrin. Tregs mediate suppression by the production of inhibitory cytokines such as IL-10 and TGF-β. IL-10 production by resident CX3CR1^hi^ macrophages contributes to expand FOXP3+ iTregs in the lamina propria (LP). gLNs, but not PP, are essential for oral tolerance development. Commensal microbiota antigens can also be transported by DCs to gLNs to induce iTregs. In addition, SIgA secreted by the plasma cells and present in the mucus blocks the adhesion of commensal bacteria and pathogens to the intestinal epithelium; it also neutralizes toxins and bacterial lipopolysaccharides that penetrate the epithelial cells.

**Figure 2.**
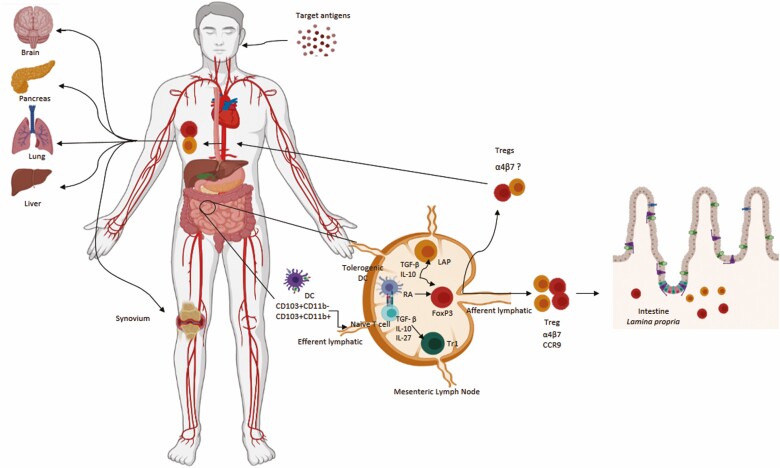
Mucosal and systemic effects of oral tolerance. In the gLNs, different types iTregs (CD4+Foxp3+, CD4+LAP+, Tr1) differentiate and acquire homing receptors such as α4β7 and CCR9/CCR10 that help them to migrate back to the intestinal lamina propria (LP) of the gut mucosa where they expand and function as regulators of gut homeostasis. It is reasonable to assume that iTregs expressing mucosal homing receptors (α4β7) and chemokine receptors upon activation would migrate through the efferent lymphatic to the thoracic duct and blood circulation to inflamed tissues throughout the body.

Despite many studies on other organs and peripheral lymph nodes, compelling evidence collected in the past ten years demonstrate that the induction of oral tolerance and its systemic effect result mostly from the action of iTregs generated in the intestinal mucosa by stimulation with antigens that reach the gut-draining lymph nodes via lymphatics [16, 20, 22, 34] (Fig. 1). In the gLNs, iTregs acquire homing receptors such as α4β7 and CCR9/CCR10 that help them to migrate back to the intestinal lamina propria (LP) where they expand under the influence of IL-10-producing CXCR3^+^ macrophages and other cells that produce both IL-10 and TGF-β [16, 35, 36]. It is not clear the mechanism by which iTregs that are generated in the gLNs can affect systemic responses, but it is reasonable to assume that these cells express homing receptors (α4β7 and chemokine receptors) upon activation that enable them to migrate to the intestinal mucosa and also to inflamed tissues throughout the body (Fig. 2). There are reports on experimental models of allergic and autoimmune diseases showing augmented frequencies of either CD4^+^CD25^+^Foxp3^+^ and CD4^+^LAP^+^ iTregs in spleen and draining lymph nodes of tolerant mice [14, 37]. In addition, clinical studies in humans show evidence of a gut-synovial axis during autoimmune inflammation in the joints. Immunization of rheumatoid arthritis patients with Influenza virus vaccine either parenterally or orally resulted in antigen-specific antibody responses by B cells isolated from enzymatically dispersed synovial tissues [38]. Immunohistochemical evaluation revealed that the mucosal-type integrin αEβ7 was detectable in samples of patients with rheumatoid arthritis and synoviocytes express its ligand E-cadherin [39]. Activated regulatory T (Treg) cells can mirror the homing abilities of effector Th1, Th2, Th17 cells allowing them to home to the sites of inflammation and act as suppressor cells there. Therefore, it is conceivable that gut-derived iTreg cells can reach chronically inflamed tissues in a similar way that activated CD4+CD25+Foxp3+ nTregs are recruited during autoimmune disease development [40].

### Microbiota and dietary components are targets and partners of oral tolerance induction

Differently from tolerance to dietary proteins that are absorbed in the proximal small intestine, it has been proposed that tolerance to gut microbiota, that are mostly located in the distal parts of the intestine (Fig. 1), does not affect systemic immune responses [16]. Studies by Duchman and coworkers showed suppression of serum IgG production against bacterial sonicates from their microbiota in humans [41] and mice [42]. On the other hand, colonic DCs cells that uptake commensal bacteria are confined within the mucosal immune system by the mesenteric lymph nodes, and that they do not induce systemic immune responses [43]. In addition, parenteral immunization of mice with 20 recombinant intestinal bacterial proteins (rIBs) generated a strong systemic IgG and CD4^+^ T cells response, whereas oral immunization with the same proteins induced secretory IgA in a T-cell independent manner but not serum IgG [44]. These findings support the concept that immune responses to gut commensal bacteria are highly restricted to the intestinal mucosa and that mice are systemically ignorant but not tolerant to their microbiota.

On the other hand, gut microbiota greatly contributes to both intestinal and systemic homeostasis by producing modulatory short-chain fatty acids (SCFA) such as butyrate, acetate and propionate, as well as organic acids like lactate. They are the main metabolites from intestinal anaerobic bacteria fermentation and bind GPR receptors (GPR43, GPR41, and GPR81) in leucocytes and endothelium inhibiting histone deacetylase (HDAC) [45]. In the intestine, SCFA stimulate secretory IgA (SIgA) production, CD8αβ ^+^ intraepithelial cell differentiation, IL-10 production by colonic resident macrophages, and Tregs generation [46]. Synergistically, lactate, as well as SCFA, inhibits the production of pro-inflammatory cytokines in myeloid and epithelial cells and displays a crucial regulatory role in the intestinal mucosa [47–49]. In addition, some strains of bacteria from the Clostridium genus enhance the secretion of local TGF-β and promote Treg differentiation in the colonic lamina propria [50, 51]. Indeed, regulatory CD4^+^CD25^+^Foxp3^+^ T cells are abundant in the colonic mucosa [52], and breakdown of these local mechanisms of immune regulation and gut homeostasis has been associated with the development of inflammatory bowel diseases (IBD) [53–55]. These studies highlight the role of gut microbiota-driven metabolites in intestinal homeostasis, but these molecules also modulate immune responses at distal sites. In murine models of allergic airway disease, for instance, SCFA and other metabolites from gut bacteria are able to shape the function of regulatory immune cells and epithelial cells in the lungs protecting mice from pulmonary inflammation [56–58]. The influence of gut microbiota and dysbiosis in extra-intestinal diseases is well documented [59] although these effects may not be directly associated with immune tolerance to gut microbiota. While the systemic benefits of oral tolerance to dietary proteins are largely mediated by antigen-specific T cells, the systemic benefits of a healthy interaction between gut microbiota and local immune system are mediated by metabolites rather than antigen-specific immune cells. Moreover, despite the reported evidence of antigen mimicry between gut bacteria and self-components as a factor interfering with some autoimmune diseases, such as uveitis [60], arthritis [61], and multiple sclerosis [62], it is not clear whether specific lineages are directly correlated with any inflammatory disease in humans.

Dietary components are also important factors in gut homeostasis conditioning the intestinal milieu towards a tolerogenic profile. Phospholipids and polyunsaturated fatty acids (PUFA) may act in the protein distribution in the lipid rafts of lymphocytes and on PPAR receptors expressed by macrophages blocking the production of inflammatory cytokines by these cells [18]. Diet containing n-3 unsaturated lipids such as fish oil or olive oil induce inhibition of NK function [63]. In addition, some micronutrients such as vitamin D and vitamin A have a direct tolerogenic effect on immune cells. Vitamin D can be metabolized by some immune cells into its active form 1,25-dihydroxyvitamin D3 (Vitamin D3) which inhibits T cell proliferation, reduces the expression of IL-2, IL-6, IL-23, IFN- γ and upregulates the production IL-10 by T cells [64]. Vitamin A is usually ingested as retinol and it be can be metabolized to retinal by alcohol dehydrogenase expressed by most cells and then from retinal to retinoic acid (RA) by retinal dehydrogenases (RALDH) expressed by certain subsets of DCs found in the gut mucosa. As further discussed in the next section, RA has the ability to induce the mucosal homing receptor α4β7 in the surface of B and T cells, to help class switch of B cells towards IgA production and also to induce the differentiation of Foxp3+ Tregs when in combination with TGF-β.[18]

## Mechanisms involved in oral tolerance induction

Although classical reports on tolerance induced by the oral route described the action of suppressor cells as critical players in the phenomenon [65, 66] studies by Weiner’s group proposed in the 1990s that oral tolerance would correlate with either active suppression or clonal anergy/anergy depending on the doses of fed antigen [67, 68]. High doses of oral antigen would direct the response towards deletion mediated by apoptosis or anergy of the specific T cells. On the other hand, lower doses would induce a differentiation of antigen-specific regulatory T cells that produce TGF-β and interleukin 4 (IL-4). However, the experiments demonstrating clonal deletion as a mechanism for oral tolerance were performed using extremely high doses of OVA (500 mg/mL) in mice bearing an OVA-reactive TCR transgene, conditions that are very distinct from physiological situations of feeding [67]. On the other hand, anergy of T cells as a mechanism to explain tolerance has become an ill-defined concept since it was demonstrated that regulatory CD4^+^CD25^+^ T cells can fit into the ‘anergic’ profile. Tregs are anergic *in vitro* and they proliferate *in vivo* upon IL-2 signaling [69, 70]. There are a number of conditions in which anergic CD4^+^ T cells function as regulatory cells *in vitro* and *in vivo* [71]. In addition, more recent work on oral tolerance showed that feeding high dose of antigen is associated with great increase in the frequency of CD4^+^CD25^+^Foxp3^+^ Treg cells [72].

### The critical action of induced regulatory T cells in oral tolerance

The role of Tregs has been established as the main mechanism for oral tolerance induction (Fig. 1). Among the Treg cells, CD4^+^CD25^+^ T cells expressing Foxp3 seem to be the most relevant ones. Thymus-derived naturally occurring regulatory CD4^+^ CD25^+^Foxp3^+^ T cells (nTregs) are abundant in the gut mucosa, as in other lymphoid organs [73]. These cells efficiently control reactivity to self-components preventing the emergency of autoimmune diseases, but they are not essential for oral tolerance induction. This was demonstrated by the intact ability of OVA-HA-double transgenic mice, which have T cells bearing OVA-reactive TCRs and B cells bearing hemagglutinin-reactive BCRs and do not generate self-reactive nTregs, to be rendered tolerant to OVA by the oral route [74]. Conversely, peripherally induced Tregs expressing Foxp3, called iTregs, are necessary for oral tolerance induction. Oral tolerance to ovalbumin could not be induced in OVA-HA-double-transgenic mice that are genetically deficient in Foxp3 and cannot produce iTregs [75]. Mucosal iTregs resemble natural thymus-derived Tregs (nTregs) but they can be distinguished in mice by the lack of expression of the transcription factor Helios [76] and/or neuropilin [77]. This was an important conclusion for the therapeutic application of oral tolerance, since the rational of using fed antigens to inhibit autoimmune diseases is that genetically susceptible individuals would have a defect in the generation of natural self-reactive Tregs in the thymus, but they could induce a peripheral counterpart of these Tregs in the gut mucosa.

The population of iTregs involved in the induction of oral tolerance is heterogeneous. Besides the classical CD4+CD25+Foxp3+ iTregs, another cell subtype of regulatory T that is abundant in the gut mucosa is the CD4^+^LAP^+^ Treg, which expresses the latency-associated peptide (LAP)/TGF-β on the surface [78]. These cells can be either FoxP3^+^ or FoxP3^-^ [78]. LAP^+^ iTregs exert their suppressive role in a TGF-β-dependent manner [79] and neutralization of these cells by intravenous administration of anti-LAP antibody prevents oral tolerance induction in murine models of experimental autoimmune encephalomyelitis (EAE) [14, 80] and arthritis [81]. TGF-β activation in these cells involves release of mature TGF-β from LAP which may occur via Glycoprotein A repetitions predominant (GARP). GARP is a transmembrane protein expressed in the surface of activated iTregs. It tethers the TGF-β/LAP complex to the cell membrane allowing the release of this cytokine during induction of Foxp3+ Treg cells in orally tolerized mice [82].

Type 1 regulatory (Tr1) cells were initially identified in humans and later in mice. They are characterized by the co-expression of CD49b and lymphocyte activation gene (LAG3) along with other regulatory markers such as PD-1, ICOS, and CTLA-4. Tr1 cells are induced by IL-27 and TGF-β secreted by DCs and they mediate their suppressive function by the secretion of IL-10 although they also produce TGF-β[83]. Their role in oral tolerance is not clear but there is a report on the development of suppressor Tr1 cells secreting both IFN- γ and IL-10 in spleens of HLA-DQ2 transgenic mice orally tolerized to deamidated gliadin [84].

It has been proposed that the main mechanism underlying the actions of mucosal iTregs is the production of inhibitory cytokines such as IL-10, TGF-β, and IL-35 [10, 85]. However, the role of anti-inflammatory cytokines in oral tolerance is a matter of debate. IL-10 is a critical cytokine for the gut homeostasis and IL-10-deficient mice develop spontaneous colitis, but these mice can be rendered tolerant by optimized protocols of antigen feeding [54, 86]. Furthermore, IL-10 production was important for the generation of OVA-specific iTreg but not for the total intestinal Tregs, whereas the presence of an intact microbiota was required for both. This suggests that oral tolerance to fed antigens and gut homeostasis are not regulated by the exact same mechanisms [87]. The role of TGF-β in oral tolerance is more documented. The generation of iTregs in the periphery is promoted by the presence of TGF-β, which can be converted from its latent and inactive form (iTGFβ) to the active cytokine (aTGFβ) through the action of the integrin αvβ8 selectively expressed by CD103^+^CD11b^−^ dendritic cells (DCs) [88]. Blocking of TGF-β through *in vivo* administration of antibodies leads to failure in the development iTregs and compromises oral tolerance induction [37]. The combined action of TGF-β and RA induces the differentiation of naive T cells into Foxp3^+^CD4^+^ T cells [89]. RA is produced by enzymatic oxidation of vitamin A by retinaldehyde dehydrogenase 2, which is expressed by these intestinal CD103^+^ DCs. The presence of RA is crucial for the development of oral tolerance. Mice with vitamin A deficiency display a poor ability to generate FoxP3^+^ iTregs cells by their CD103^+^CD11c^+^ DCs and they are not able to develop tolerance after ingestion of OVA by gavage [90] or by OVA-containing breast milk when they are neonates [91]. Conversely, vitamin A supplementation in the diet of mice throughout life induces an increase in the frequency of CD4^+^Foxp3^+^ Tregs that also express surface expression of TGF-β [92].

### Antigen-presenting cells in oral tolerance induction

The interactions between antigen-presenting cells (APC) and naive T cells precede the development of tolerance and immunity *in vivo*. APCs, including DCs and macrophages, have been described as critical in triggering iTreg cell differentiation [93]. The expression of CX3CR1^+^ has been studied as a marker for tolerogenic DCs and macrophages in the LP. Targeted deletion of MHCII^+^ CX3CR1^+^ cells abrogates oral tolerance to OVA although CX3CR1-deficient mice show intact tolerance [87]. A study using cell-transfer strategies demonstrated that CX3CR1^+^ macrophages expressing CD11b are important in gut homeostasis and in the proliferation, but not in the induction of LP CD4^+^Foxp3^+^ iTregs [94]. These cells can transfer captured antigens to CD103^+^DCs via a Connexin-43 (gap junction - Cx43) [95] and CD103^+^ DCs derived from the LP migrate to mLNs where they produce large amounts of RA and TGF-β [89, 94] inducing the differentiation of iTregs. Therefore, CD103^+^ cDCs seem to be the direct inducers of iTregs, whereas CX3CR1^+^ macrophages have a role in antigen uptake and later expansion of iTregs in the gut mucosa [16].

It is plausible that luminal protein antigens reach the draining gLNs by two ways: (1) carried by CD103^+^CCR7 cDCs as demonstrated by some studies [22, 96]; (2) in a cell-free manner carried in chylomicrons through the lymphatic vessels as shown by a recent study [20]. Indeed, there are reports showing that LP cDCs expressing the chemokine receptor 7 (CCR7) migrate via afferent lymphatics to mLN where local events drive the systemic consequences of tolerance [96. Either the impairment of DC migration by genetic deletion of CCR7 or the surgical removal of mLN results in failure to establish oral tolerance [22]. Lesions in the lymphatic vessels caused by infections also interfere in the migration of luminal antigens and of CD103+ dendritic cells from the gut mucosa to the mLN compromising the generation of oral tolerance [97].

### Other lymphoid cells involved in oral tolerance

Although regulatory CD4+ T cells seem to be the main players, other cell types participate in the generation and maintenance of tolerance in the intestine (Fig. 2).

Natural killer T cells (NKT) are highlighted by some authors as important cells in oral tolerance induced with haptenized colon proteins or alloantigens [98, 99]. These cells express the Fas ligand and produce high levels of IL-4, they can participate in apoptosis of activated T cells and they may induce the conversion of T_H_1 to T_H_2 cells. Moreover, the state of tolerance can be transferred by liver NKT cells [98, 99]. The role of NKT cells in oral tolerance is not undisputable. Another report suggests that although NKT cells regulate the balance between T_H_1 and T_H_2 in response to dietary antigens, oral tolerance can be established in mice lacking these cells due to a genetic deficiency of the Jα 281 component of the invariant TCR [100].

γδ T cells are abundant in the intestinal epithelium and they are not dependent on MHC signaling responding quickly to luminal antigens through the secretion IFNγ and IL-17. They also have immunoregulatory functions, helping to preserve the integrity of the epithelial surface during intestinal inflammation [101]. Some studies show that IL-10 production and oral tolerance induction are compromised in mice depleted of γδ T cells [102] and in mice genetically deficient in these cells [103]. A unique subset of γδ T cells that express LAP (TCRγδ ^+^LAP^+^) also seem to have a role in controlling intestinal inflammation. These cells are found in the Peyer patches and in the small intestine LP, they present antigens and, although they do not express Foxp3, they are capable of inducing CD4^+^Foxp3^+^ Treg cells. Transfer of TCRγδ ^+^LAP^+^ cells ameliorate both colitis induced by the transfer of CD4^+^ CD45RB^high^ cells to immunodeficient mice (a rodent model for Crohn’s disease) and DSS-induced colitis by expanding Foxp3+ Treg cells [101].

Some classical studies suggest that CD8^+^ T cells participate in the induction of oral tolerance [66, 104], and that IL-4 or IL-10 production can be activated by a population of regulatory CD8^+^ T cells, even when cytotoxic CD8^+^ T cells are inhibited by oral administration of antigen [105]. More recently, human and mouse regulatory CD8+ T cells expressing lower levels of Foxp3 [106], as well as a population of CD8+ T cells bearing surface LAP and displaying suppressive properties have been described [107]. There is experimental evidence that CD8+ T cells participate in the suppression of EAE in mice by oral administration of MBP [108], and defects in colonic lamina propria CD8+ T cells in IBD patients [109]. In less recent studies, the function of CD8^+^ T cells was evaluated in mice deficient in genetically modified CD8^-^ or β2m [110, 111] and in mice treated with anti-CD8 antibodies [108, 112]. These studies indicate that of CD8^+^ T cells may contribute but they are not essential for the induction or maintenance of oral tolerance.

Innate lymphoid cells (ILCs) are also known to participate in protective immunity and regulation in homeostasis and inflammation. These cells are abundant in the intestinal mucosa and it is plausible that they participate in oral tolerance development by conditioning the intestinal milieu towards a tolerogenic profile. ILC2s have a critical role in allergic and anti-helminth responses but they also secrete amphiregulin, a molecule involved in tissue repair [113]. ILC3s secrete IL-22, a cytokine important for mucosal homeostasis, and granulocyte-macrophage-colony factor (GM-CSF), a factor that promotes the production of tolerogenic molecules such as RA and IL-10 by gut tolerogenic DCs [114]. However, in pathological conditions, these same cells may have pro-inflammatory effects. Studies show that ILC3-derived GM-CSF can promote colitis [115] and ILC3-derived IL-22 and IL-17 aggravate IBD in humans [116]. Therefore, it appears that ILC-derived cytokines are important in regulating tolerance and inflammation and further studies need to be done to better understand their role in oral tolerance.

## Clinical issues involved in the therapeutic application of oral tolerance

Our group and others have demonstrated that oral tolerance, as an antigen-specific OIT, is successfully induced in several models of inflammatory conditions (Table 1) and also in many clinical studies in humans (Table 2). From this extensive work performed by different groups, some important issues concerning the clinical application of oral tolerance emerged: (1) the target antigen to be used for oral administration is not always known in many inflammatory diseases; (2) the lower efficacy of oral tolerance for already sensitized individuals; (3) oral tolerance requires large doses of antigen; and (4) the age of first feeding affects susceptibility to oral tolerance induction.

**Table 1.** Experimental models of diseases suppressed by oral tolerance

Model	Immunizing Ag	Oral Ag	Prevention or treatment
Allergic asthma	Der p 1 (45–145) Tg rice	Der p 1 (45–145) Tg rice	Prevention
Arthritis	Collagen type II chicken	APL6 Tg rice; Hsp65-Producing *L. lactis*	Prevention
Arthritis	mBSA	Collagen II; mBSA	Prevention
Atherosclerosis	Cholesterol, lard and cholate	Hsp65-Producing *L. lactis*	Prevention
Atherosclerosis	*M. Tuberculosis*	Hsp65	Prevention
Colitis	CD4^+^ CD45RB^high^ T cell transfer	OVA	Prevention
Colitis	DSS	Hsp65-Producing *L. lactis*	Prevention
Colitis	TNBS	OVA	Prevention
Diabetes	LCMV	Insulin	Prevention
Diabetes	None (NOD Mice)	BLPs-SCI-59; CTB-insulin; GAD; Insulin β chain peptide 10–24 + IL-10; Proinsulin + IL-10 + anti-CD3	Prevention
EAE	MBP	MBP + IL-10	Prevention
EAE	MBP	MBP	Prevention and Treatment
EAE	MOG	Hsp65-Producing *L. lactis*	Prevention
EAE	MOG	MOG + IL-10	Treatment
EAE	PLP	MBP	Prevention
EAN	P2-peptide	P2-peptide	Prevention
EAU	IRBP peptides	IRBP peptides	Prevention and Treatment
EAU	S-Ag	HLA peptide; S-Ag	Prevention
Food allergy	OVA	OVA	Prevention
GVHD	Splenocytes	Spleen protein extract + *L. lactis* NCDO2118	Prevention
Myasthenia gravis	TAChR	IRT5 probiotics	Prevention
Myasthenia gravis	TAChR	AchR	Prevention and Treatment
Nerve injury	None	MBP	Prevention
Nickel and chromium sensitization	K_2_Cr_2_O_7_ and NiSO_4_	Nickel and chromium	Prevention
Sjögren’s syndrome	Ro peptides	Ro peptides	Prevention
Stroke	MBP	MBP	Prevention
Thyroiditis	Thyroglobulin	Thyroglobulin	Prevention
Transplantation	None	Class I MHC antigens (RT1.A)	Prevention
Wheat allergy	Gliadin	Gliadin	Prevention

AchR, acetylcholine receptor; BLPs, bacterium-like particles; EAE, experimental autoimmune encephalomyelitis; EAN, experimental autoimmune neuritis; EAU, experimental autoimmune uveitis; GVHD, graft-versus-host disease; IRBP, interphotoreceptor retinoid-binding protein; LCMV, lymphocytic choriomeningitis virus; *L. lactis*, *Lactococcocus lactis*; MBP, myelin basic protein; MHC, major histocompatibility complex; MOG, myelin oligodendrocyte glycoprotein; OVA, ovalbumin; SCI, single-chain insulin.

**Table 2.** Successful clinical studies using oral tolerance as immunotherapy

Disease	Oral Ag
Autoimmune Thyroid Disease	Thyroglobulin and thyroid peroxidise [117]
Cow’s Milk Protein Allergy	Cow’s milk [118,119]
Cow’s Milk Protein Allergy	Hydrolyzed cow’s milk protein-based formula [120]
Other allergic manifestations in children with cow’s milk allergy	Hydrolyzed casein formula containing *Lactobacillus rhamnosus* GG [121]
Dust mite allergy	Dermatophagoides pteronysstnus [122,123]
Egg Allergy	Eggwhite powder [124]
Multiple sclerosis	Bovine myelin (contains MBP and PLP) [125,126]
Peanut allergy	Peanut (oral immunotherapy) [127,128]
Rheumatoid arthritis	Bovine Colagen II [129]
Rheumatoid arthritis	Peptide dnaJP1 [130]
Systemic nickel allergy	Nickel [131]
Uveitis	HLA-peptide B27PD [132]
Uveitis	S antigen (S-Ag) [133]
Uveitis due to Behcet’s disease	Behcet’s disease-specific peptide p336–351 [134]

MBP, myelin basic protein; PLP, proteolipid protein; S-Ag, S antigen.

### Target antigens for oral tolerance

Considering that the therapeutic use of oral tolerance includes the specific suppression of immune responses to inflammatory reactions, an important issue is the identification of the antigens involved in the pathological process. Sometimes it is not clear which are these target antigens. Fortunately, oral tolerance spreads the regulatory events induced to other antigens that are presented in the same environment or context during the inflammatory reaction to be suppressed. This phenomenon was first described by Vaz and coworkers [135] as cross-suppression, later renamed as bystander suppression by Weiner’s group [136] or as indirect effects of oral tolerance by Carvalho and coworkers [137]. It correlates well with the observation that during the course of inflammatory diseases in animals and humans, there are reactivities to multiple antigens in the target organ [138]. This spread of reactivity has been observed for multiple sclerosis, arthritis, diabetes, and autoimmune thyroiditis, for instance, diseases in which several tissue target antigens have been identified.

Moreover, even in other inflammatory conditions such as atherosclerosis where the target antigens are not known, commonly expressed molecules in inflammation such as heat shock proteins can be used as antigens for oral tolerance induction [139]. Bystander suppression is defined as a suppressor activity mediated by antigen-specific regulatory T cells generated after oral administration of antigen that can deliver non-specific factors, which is capable of mediating suppression of immune responses for antigens presented along with the fed antigen. After the cross-suppression model described in 1981 using two unrelated antigens, OVA and KLH, bystander suppression was reported when EAE was prevented in mice that were fed MBP and co-immunized with MBP and proteolipid protein (PLP) [136]. The indirect effects of oral tolerance induction were demonstrated using OVA-fed mice in which granuloma reaction caused by the helminth *Schistosoma mansoni* and cutaneous scar formation were inhibited by simultaneous injection of OVA at the time of infection/inflammation induction [140, 141]. In addition, our group has also used a recombinant Hsp65-producing *Lactococcus lactis* to prevent and treat inflammatory disease models such as EAE [14], DSS-induced colitis[142] and antigen-induced arthritis [81] showing that Hsp65-induced Tregs are able to inhibit inflammatory reactions towards myelin antigens, colonic antigens and collagen. It has been proposed that bystander suppression is due to the simultaneous presentation of unrelated antigens by the same APC in the draining lymph node [10]. However, there is also evidence that the indirect effects of oral tolerance occur when the two antigens are injected at distinct sites and even when they were administered up to 72 hours apart [137]. It is plausible that Tregs do not have to be specific for the one target antigen, but they can suppress inflammation in the tissue or organ as long as they recognize any antigen in the environment. Although the mechanisms involved in bystander suppression/indirect effects of oral tolerance are still a matter of debate and they have not been demonstrated in clinical studies yet, the strategy of cross-suppression may theoretically help to circumvent the need for a well-defined target antigen for oral tolerance induction in human inflammatory diseases as well.

### Oral tolerance induction after sensitization

A second important issue to be solved for clinical application of oral tolerance is the fact that feeding antigens are very efficient in naive but not in primed animals. There are a number of studies showing that oral administration has been effective only when given before disease induction [10, 143]. It is possible that the very mechanism of suppression generated by oral tolerance is due to the earlier appearance of antigen specific T cells that are generated by feeding, before effector T cells take the stage [37]. However, the use of adjuvants and regimens of feeding can positively modulate the mechanisms of oral tolerance. Among the adjuvants, the use of TGF-β and dimaprid (a histamine type 2 receptor agonist) was reported to help inhibition of collagen-arthritis even after disease onset [144]. Coupling Hsp-60 peptides to the subunit B of cholera toxin also potentiates oral tolerance to uveitis [145]. Oral co-administration of cytokines such as IL-4, IL-10, IFN-τ, and IFN-β, as well as some antigenic products of parasites such as *Schsitosoma mansoni* and *H. polygyrus,* synergize with antigen feeding to enhance suppression [10]. Combined oral administration of the probiotic strain *Lactococcus lactis* NCDO2118 and donor splenocytes was also able to strongly inhibit GVHD disease in mice [146]. Other combination OIT that have been reported to successfully boost the effects of oral tolerance to specific antigens are anti-inflammatory cytokines such as IL-4 and IL-10 [11, 12] as well as oral delivery of anti-CD3 monoclonal antibodies [13].

Frequency and regimens of feeding are also critical determinants of oral tolerance induction [10]. We and others have previously demonstrated that the continuous administration of antigen in the drinking water is more effective for tolerance induction and more lasting than a single dose or multiple doses of antigen administered through the intragastric route by gavage [147–149]. It is possible that continuous delivery of antigens resembles a natural process that evolved to trigger tolerogenic signals by APCs in the intestinal mucosa [149]. Some strategies that mimic this process, such as multiple emulsion [150] and liposome systems [151], seem to improve oral tolerance to collagen-induced arthritis and proliferative responses. In the same line, a recent developed recombinant Hsp65-producing *Lactococcus lactis* NCDO2118 is a slow-delivery strategy used by our group to prevent and control inflammatory diseases. This recombinant lactic bacteria lodges in the duodenum and induces high frequencies of CD4^+^Foxp3^+^ and CD4^+^LAP^+^ iTreg in mesenteric lymph nodes that can be found also in distant peripheral lymphoid organs [14, 81, 142]. The anti-inflammatory probiotic properties of lactic bacteria such as *L. lactis* combined with its ability to promote the slow delivery of antigen in a highly tolerogenic segment of the intestine may render these bacteria a potential efficient alternative adjuvant for antigen-specific OIT. In addition, the continuous feeding protocol inhibited airway inflammation in mice already sensitized to OVA up to 7 days after priming [74]. Thus, adjuvants and optimal protocols of antigen delivery can be used to improve oral tolerance induction in a clinical setting.

### Doses of antigen for oral tolerance induction

Due to the enzymatic digestion of proteins in the gastro-intestinal tract, doses of antigen used to induce oral tolerance are usually large, and this is another constraint when designing protocols for clinical studies and for its therapeutic use. However, the disease to be treated may also be an important variable to consider in this regard. Some studies demonstrated that oral administration of low doses of allergens promotes allergic desensitization. Burks and coworkers showed in a double-blind, placebo-controlled, and randomized egg allergy study that 75% of participants were desensitized at 22 months and 28% had sustained unresponsiveness using low-dose egg white powder [124]. For milk allergy, desensitization treatments have been widely reported using low doses of antigen and these protocols have already been adopted in clinical practice. Yanagida and coworkers [118] also demonstrated that oral treatment using 3 ml milk for 1 year resulted in unresponsiveness in 58.3% of patients while only 33.3% had diminished responses when treated with 25 ml. Using nasal and sublingual routes is an interesting alternative to address this specific caveat since they require much lower doses of antigen to trigger tolerogenic mechanisms.

### The good age for oral tolerance induction

Age is another important factor that should be taken into account. Although the first antigen exposure occurs early after birth, neonates are refractory to oral tolerance induction [152]. This resistance may be explained by the fact that neonates display deficiencies in protein degradation by proteolytic enzymes, and in antigen presentation due to a physiological deficiency of vitamin A [153]. Neonates have an immature immune system, undeveloped gut anatomy and metabolism [153]. These deficiencies can be circumvented by using either oral peptides [154] or introducing the antigens via lactation [155, 156]. In a series of studies, Verhasselt’s group showed that early administration of allergens during breast feeding provides two modulatory strategies for oral tolerance enhancement: first, lactation would resemble a continuous feeding protocol; second, breast milk contains immune mediators that are capable of compensating for the neonatal deficits and of assisting tolerance induction [157]. This raises the possibility that early exposure of neonates to allergens through breast milk or even placental transfer [158] might be a way of inducing tolerance instead of sensitization for allergic diseases, a proposition still under debate.

In line with these findings in mice, some reports suggest that an important period for oral tolerance induction comprises the phase prior to the introduction of solid foods [156]. Clinical studies have demonstrated that early consumption of antigens through the oral route has a beneficial effect. Katz and co-workers have shown that early introduction of cow’s milk protein has a protective effect against IgE-mediated cow’s milk allergy. Also, Tan and coworkers [159] showed in a randomized trial that the contact with egg at four months of age reduced the levels of allergic sensitization and promote tolerance instead. However, a concern still exists that early antigen consumption may be related to food sensitization and allergy. Rekima and collaborators showed that early oral exposure to *Dermatophagoides pteronyssinus,* a house dust mite allergen through breast milk, favored the development of immunological events such as the induction of a Th2 response to OVA, an unrelated antigen. This study suggests that this contact can unbalance immune response and trigger food allergy instead of oral tolerance. The same group conducted a study with a human cohort and demonstrated that *D. pteronyssinus* exposure through maternal milk represents a risk for sensitization, which brings a word of caution in recommendation for early exposure to oral antigens [160].

In addition to early antigen exposure, another factor that interferes with oral tolerance induction is the aging process. Several immune changes are related with aging. Due to thymic involution, the T cell repertoire is less diversified, naive T cell frequency decreases and there is an increase in memory T cells. Bone marrow production of B cells is also affected by aging [161]. Our group has already described that aging is associated with the impairment in oral tolerance induction in mice [162], and that old animals present reduction in the frequencies of regulatory-type TCRγδ+ intraepithelial lymphocytes (IELs), and diminished levels of TGF-β and IL-10 [163] in the gut mucosa. Others have reported reduced production of secretory IgA in aged mice [164]. On the other hand, humans preserve their ability to produce CD4+CD25 +Foxp3+ iTreg during inflammatory and infectious diseases [165]. Interestingly, aged mice that are refractory to oral tolerance induction by gavage can be effectively rendered tolerant by continuous feeding of antigen confirming the robustness of this protocol of feeding [147, 148].

## Clinical applications of oral tolerance

Although we described some issues concerning oral tolerance induction, several groups have published studies using oral tolerance as immunotherapy with variable degree of success depending on the disease and on the protocol of feeding (Table 2).

### Oral tolerance application in food allergy

It is already described that failure in oral tolerance induction or its breakdown results in food allergy, an important public health problem in developed countries. As a result of an increase in IgE levels, the most common symptoms of food allergy are skin disturbances, as well as gastrointestinal, respiratory and cardiovascular alterations. Severe food allergy can result in anaphylaxis involving several organ systems and may compromise respiratory tract, inducing life-threatening reactions [166]. Food allergy affects about 5% of adults and 8% of children in westernized countries [167]. Susceptibility of children to develop food allergy may be related to the high intestinal permeability caused by the immature development of the intestinal mucosa barrier and increasing exposure to intact proteins that can lead to sensitization [168]. In addition, the SIgA system is not fully developed until the age of four years old, so both the immune and physiological immaturity of the mucosal barriers may be related to the prevalence of gastrointestinal infections and food allergies in the first years of life [169]. In this scenario, oral tolerance protocol can be a suitable strategy to desensitize allergic children, and to treat allergic diseases in adult individuals.

In the past 10 years, OIT has been extensively tested for food allergy as a more efficacious and lower-risk immunotherapy than subcutaneous desensitization. Single-allergen OIT for treatment of IgE-mediated food allergy has shown efficacy to modulate food allergy to the main dietary allergens, egg, milk, and peanut [170]. Therapeutic outcomes following OIT includes desensitization, which is the elimination of clinical reactivity to the allergen while in active therapy, and sustained unresponsiveness which is defined as the elimination of this reactivity after cessation of the treatment. Usually phase 1 in OIT includes a scalation of micrograms of food allergenic protein to milligrams in one or two days. The next building up phase includes an increment of once- or twice-a-week dose until reaching a maintenance dose [170]. It is still under debate whether the unresponsiveness observed in OIT accomplishes only a transient state of desensitization that is dependent on a constant antigen exposure, or whether it can attain a more sustained effect even after antigen withdrawal. It seems that sustained unresponsiveness is accomplished only in 10–15% of the individuals [170].

OIT was successfully tested in children who were allergic to egg proteins. However, when evaluating the capacity to maintain the tolerance state, only 1/3 of children in the study could maintain it after 3 months of food withdrawal [171]. Oral tolerance treatment was also tested in children of 5 to 17 years old with severe milk allergy, using a protocol with increasing doses of antigen consumption. The study achieved a significant number of tolerated subjects (36%), who were able to consume cow’s milk and dairy products after 1 year and 50% of them had a partial tolerance result, which enabled higher amount of cow’s milk intake when compared with the control allergic group [172]. However, clinical trials using unprocessed antigen can enhance the risk of adverse reactions that would require the use of epinephrine. Meta-analysis studies demonstrate that OIT approach, although inducing desensitization, is related with considerable increase in anaphylactic reactions over placebo or avoidance controls [173]. In order to circumvent this problem and to enable safer protocols of desensitization, the use of hydrolyzed protein-based formulas have presented favorable results. Considering that this formula possesses low allergenicity, a double-blind, randomized study was conducted with 25 children between 1and 9 years old consuming partially or extensively hydrolyzed formulas. The study reported that OIT was capable of increasing the amounts of milk tolerated by the children without causing systemic symptoms [120].

### Oral tolerance as alternative treatment for autoimmune diseases

The induction of oral tolerance has also been widely studied as an alternative for autoimmune disease prevention or treatment. It has been shown that daily intake of insulin capsules (7.5 mg) by children and adolescents with islet cell autoantibodies improved their metabolic parameters, evidenced by a better response to the oral glucose tolerance test (OGTT), suggesting a positive effect of the oral immunotherapy [174]. However, the same treatment does not prevent or delay the onset of type 1 diabetes (T1D) [175, 176]. Results on NOD mice, which spontaneously develop T1D, are controversial. Some studies show that oral administration of insulin or its B-chain peptide to these ice delay the onset of this disease and decreases insulitis [177, 178]. A more recent study reported that NOD mice present several alterations in the gut mucosa, such as reduced levels of SIgA and mucus, bacterial translocation to the pancreatic lymph nodes, altered frequencies of inflammatory dendritic cells in the mesenteric lymph nodes and a lower frequency of Tregs in the duodenal and jejunal-draining lymph nodes. These defects may explain their inability to develop tolerance even upon continuous feeding of OVA [179]. It would be interesting to investigate whether these mucosal alterations are also present in humans with or at risk to develop T1D to explore one of the many possible reasons why oral immunotherapy failed to prevent or delay the onset of this disease in some clinical trials.

In 1993, Weiner and collaborators treated patients with multiple sclerosis for one year with 300 mg of bovine myelin that contains MBP and PLP similar to human myelin. These patients had less attacks than patients who received placebo. In addition, the T cells of patients treated with bovine myelin do not proliferate when stimulated *in vitro* with MBP and PLP [125]. In a subsequent study using the same protocol, T cells of patients treated with bovine myelin showed an increase in the secretion of TGF-β1 in response to MBP and PLP, while no change was observed in the secretion of IFN-γ [126]. However, it is not clear whether the patient’s gender or the MHC II phenotype was related to the effectiveness of the treatment, since all eight male patients who received bovine myelin did not have the HLA-DR2, while six of the seven women in the placebo group had the HLA-DR2 [125]. The improvement of uveitis was also observed in patients who received S antigen orally [133] or HLA-I B27PD peptides that mimic S antigen [132] with improvement in visual acuity [132, 133] and decrease in inflammation [132].

Some studies have also evaluated the use of different antigens and dosages for the treatment of rheumatoid arthritis. Koffeman and collaborators (2009) [130] tested the oral administration of 25 mg dnaJP1 peptide in rheumatoid arthritis patients for 6 months reporting a reduction in TNF-alpha-producing T cells and an increase in IL-10-producing Treg cells associated with amelioration of disease. Barnett *et al*. (1998) evaluated four different dosages of orally administered bovine collagen II (CII) in patients with rheumatoid arthritis. After 24 weeks of treatment, the group that received the lowest dose of collagen (20 µg/day) presented the highest improvement in clinical parameters. In another study, treatment with 0.5 mg/day bovine CII showed to be the ideal dose to improve the clinical parameters of the disease [129, 180]. On the other hand, administration of doses of 0.1 mg/day for 1 month, 0.5 mg/day for 5 months, or 1 mg/day and 10 mg/day for 12 weeks of bovine collagen resulted in no statistical improvement of rheumatoid arthritis [181, 182].

A major challenge in the clinical studies of mucosal tolerance for autoimmune diseases is the interference of systemic immunosuppressive drugs that are commonly used to control symptoms and achieve remission. Administration of corticosteroids or nonsteroidal anti-inflammatory drugs highly interferes with the generation of regulatory T cells [183]. In some oral immunotherapy clinical trials for autoimmune diseases, patients are allowed to take low doses of steroids during the treatment protocol [130, 180] and this may impair the achievement of the desired outcome. Even though patients are asked to discontinue the use of immunosuppressive medication in some studies, the chronic use of these drugs prior to the treatment protocol might have long-lasting effects and impose barriers to oral tolerance induction. Moreover, patients may not tolerate the discontinuation of immunosuppressive drugs for a long period of time in clinical trials, especially if they are in a placebo group.

## Conclusions

Oral tolerance is a physiological phenomenon that protects the body from inflammatory reactions against harmless natural antigens such as dietary proteins and microbiota. It has been extensively and successfully tested in many disease models and in human clinical trials as an effective way to deliver tolerogenic signals and to induce robust, long lasting, specific suppression. Oral tolerance as an antigen-specific type of OIT is devoid of the side effects of classical immunosupression currently used as immunotherapy and it is well suited for chronic use. However, there are still few critical issues to be solved before its therapeutic application reaches the ordinary clinical practice. Addressing these issues experimentally and in the clinical setting is pivotal to make it a successful immunotherapy.

## Data Availability

There are no novel data associated with this review article.

## Abbreviations

APC: Antigen-presenting cells
CCR7: Chemokine receptor 7
DCs: Dendritic cells
EAE: Experimental autoimmune encephalomyelitis
Foxp3: Forkhead box p3
GALT: Gut-associated lymphoid tissue
GARP: Glycoprotein A repetitions predominant
gLN: Gut-draining lymph nodes
IBD: Inflammatory bowel diseases
ILCs: Innate lymphoid cells
iTreg: Induced regulatory T cells
LAP: Latency-associated peptide
MBP: Myelin basic protein
mLN: Mesenteric lymph nodes
NKT: Natural killer T cells;
nTregs: Natural thymus-derived Tregs
OIT: Oral immunotherapy
OVA: Ovalbumin
PLP: Proteolipid protein
PP: Peyer patches
RA: Retinoic acid
RALDH: Retinal dehydrogenases
RORγt: RAR-related orphan receptor gamma t
SCFA: Short-chain fatty acids
SIgA: Secretory IgA
T1D: Type 1 diabetes
TGF-β: Transforming growth factor β
Treg: Regulatory T cells
